# Genome Sequence of *Geothermobacter* sp. Strain EPR-M, a Deep-Sea Hydrothermal Vent Iron Reducer

**DOI:** 10.1128/genomeA.00424-17

**Published:** 2017-06-08

**Authors:** Benjamin Tully, Pratixaben Savalia, Karla Abuyen, Christina Baughan, Eric Romero, Cynthia Ronkowski, Brandon Torres, Jason Tremblay, Anthony Trujillo, Madeline Tyler, Ileana Pérez-Rodríguez, Jan Amend

**Affiliations:** a Department of Biological Sciences, University of Southern California, Los Angeles, California, USA; b Center for Dark Energy Biosphere Investigations, University of Southern California, Los Angeles, California, USA; c Department of Earth Sciences, University of Southern California, Los Angeles, California, USA; d Community College Cultivation Cohort, University of Southern California, Los Angeles, California, USA

## Abstract

*Geothermobacter* sp. strain EPR-M was isolated from a hydrothermal vent on the East Pacific Rise and has been shown to participate in the reduction of Fe(III) oxides. Here, we report its 3.73-Mb draft genome sequence.

## GENOME ANNOUNCEMENT

Hydrothermal vent systems are sources of reduced compounds to the deep ocean, including metals (1). When introduced to the oxic bottom waters, reduced metals, specifically iron and manganese, are oxidized through abiotic and biotic mechanisms and precipitate out of the water column (2). Organisms from the *Geobacteraceae*, including the thermophilic *Geothermobacter ehrlichii*, isolated from a hydrothermal vent on the Juan de Fuca Ridge (3), have a documented role in the reduction of Fe(III) oxides in many environments (4, 5). Here, we present the genome sequence of *Geothermobacter* sp. EPR-M, a mesophilic strain isolated from the geographically distant hydrothermal system at 9°50′N on the East Pacific Rise.

Genomic DNA from strain EPR-M was extracted using the UltraClean microbial DNA isolation kit (Mo Bio, Inc., Carlsbad, CA), according to the manufacturer’s protocol, and then sequenced using an Illumina HiSeq 2500 2 × 150 bp platform at the University of Southern California Genome Core. A total of 135,882,866 raw paired-end sequences were generated. Sequences were processed using Trimmomatic version 0.33 (6) to remove adapter sequences, areas of pronounced k-mer content skews in the first 10 bp and last 15 bp, and regions with low quality scores (*Q* < 28). A total of 105,006,698 high-quality paired sequences were *de novo* assembled using SPAdes version 3.7.1 (7), generating 9,895 contigs. Contigs representing strain EPR-M were identified based on estimated coverage values, with the target genome consisting of 149 contigs at >1,000× coverage and the remaining contigs representing carryover DNA at ~1× coverage. Only the 59 contigs ≥1 kb are included in the presented genome. The *Geothermobacter* sp. strain EPR-M genome is 3.73 Mb in length, with an *N*_50_ value of 140,284 bp and a G+C content of 59.4%. Annotation was performed using the NCBI Prokaryotic Genome Annotation Pipeline (version 4.1), resulting in 3,264 coding DNA sequences (CDSs), a coding density of 88%, 6 rRNAs (2 copies each of the 5S, 16S, and 23S genes), and 50 tRNAs. Based on results from CheckM version 1.0.3 (8), the final genome is estimated to be 99.35% complete based on the presence of 245 of 247 markers from the lineage *Deltaproteobacteria* and 0.65% “contaminated” based on the presence of a duplicate copy of a single marker gene.

Genomic analysis has revealed that strain EPR-M contains genes encoding several metabolic pathways that have been observed in the cultured isolate. Genes coding for the respiration of hydrogen (*hyaAB* and *hydAB*) and various organic substrates (formate, acetate, and lactate) were detected, along with those with the potential to use nitrate (*narGH*) as a terminal electron acceptor. Several key genes were not observed for metabolic processes that have been observed in culture. Even though the reduction of Fe(III) oxides has been observed, genes encoding this process (such as *mtrABC*) could not be identified, although the genome does possess 14 cytochromes for which functions are not assigned. Further, EPR-M has been observed to grow under autotrophic conditions, but it lacks the genes necessary to complete the known carbon fixation cycles/pathways (9).

### Accession number(s).

This whole-genome shotgun project has been deposited at DDBJ/ENA/GenBank under the accession no. NAAD00000000. The version described in this paper is version NAAD01000000.
